# From the Editorial Board

**DOI:** 10.2188/jea.17.215

**Published:** 2007-12-19

December 31, 2007 is the last day as the Editor-in-Chief of the Journal of Epidemiology for me. The reason why I can serve as the Chief for the past 6 years is the support of the Editorial members, and I appreciate their help. With their participation, the average and median of 23 days between receiving the first submission of a paper and returning the Editorial Comments to the authors has achieved. If they were not the members of the Editorial Board, each of them could have written their own scientific article 2 or more a year! Such a tough work has been to be the member for the past 6 years. I also thank reviewers. Reviewing an article within 2 weeks is also tough work, but this short-period reviewing system support the 23 days as well.

During the 6 years, the Journal progressed in several points. First, the Journal started to be cited by the Thomson ISI. I do really not know the evaluation of the impact factor between 1.24 and 1.25, but this is expected to introduce many submissions to the Journal. Second, the Journal became to be able to be read from all over the world through the net. This may induce the citations of the article on the Journal to other papers. Also, submission of a paper through e-mail is now possible.

Although the current Board is responsible for articles received till the end of 2007 through the decision of accept or reject, I will retire soon. In and after 2008, I will support the more progress of the Journal as a Board member of the Japan Epidemiological Association as well as one of the members of the Association. I hope Dr. Tomotaka Sobue, the next Editor-in-Chief, and his colleagues progress the Journal drastically.

Thank you all.

Yosikazu Nakamura, MD, MPH, FFPH
Editor-in-Chief, Journal of Epidemiology

